# (1*S*,3*R*,8*R*)-2,2-Dibromo-3,7,7,10-tetra­methyl­tricyclo­[6.4.0.0^1,3^]dodec-9-ene

**DOI:** 10.1107/S1600536812032333

**Published:** 2012-07-21

**Authors:** Ahmed Benharref, Lahcen El Ammari, Essêdiya Lassaba, Najia Ourhriss, Moha Berraho

**Affiliations:** a Laboratoire de Chimie des Substances Naturelles, "Unité Associé au CNRST (URAC16)", Faculté des Sciences Semlalia, BP 2390 Bd My Abdellah, 40000 Marrakech, Morocco; b Laboratoire de Chimie du Solide Appliquée, Faculté des Sciences, Avenue Ibn Battouta BP 1014 Rabat, Morocco

## Abstract

The title compound, C_16_H_24_Br_2_, was synthesized from β-himachalene (3,5,5,9-tetra­methyl-2,4a,5,6,7,8-hexa­hydro-1*H*-benzocyclo­heptene), which was isolated from the essential oil of the Atlas cedar (*Cedrus Atlantica*). The mol­ecule is built up from two fused six- and seven-membered rings and an additional three-membered ring from the reaction of β-himachalene with dibromo­carbene. The six-membered ring shows a screw-boat conformation, whereas the seven-membered ring displays a boat conformation; the dihedral angle between the mean planes through the rings is 57.9 (4)°. The absolute structure was established unambiguously from anomalous dispersion effects.

## Related literature

For the isolation of β-himachalene, see: Joseph & Dev (1968 ▶); Plattier & Teiseire (1974 ▶). For the reactivity of this sesquiterpene, see: Lassaba *et al.* (1997 ▶); Chekroun *et al.* (2000 ▶); El Jamili *et al.* (2002 ▶); Sbai *et al.* (2002 ▶); Dakir *et al.* (2004 ▶). For its biological activity, see: Daoubi *et al.* (2004 ▶). For conformational analysis, see: Cremer & Pople (1975 ▶).
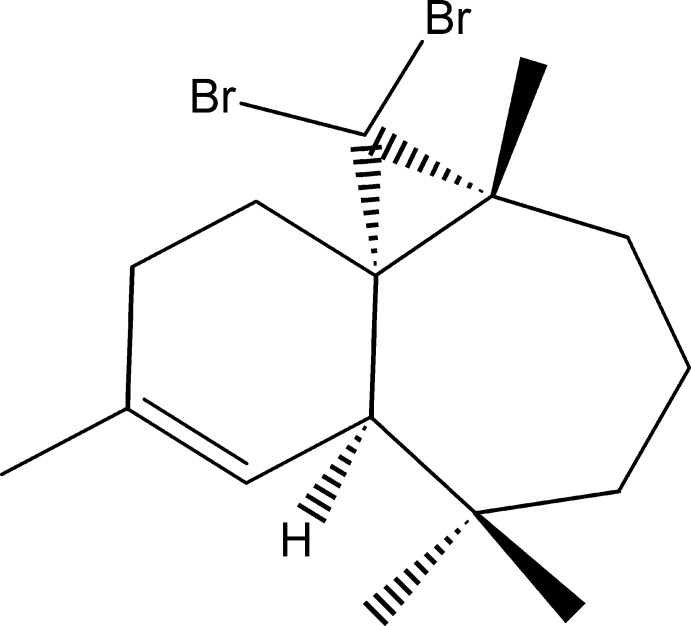


## Experimental

### 

#### Crystal data

C_16_H_24_Br_2_
*M* *_r_* = 376.17
Orthorhombic, 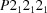

*a* = 9.7464 (14) Å
*b* = 12.1633 (16) Å
*c* = 13.5352 (18) Å
*V* = 1604.6 (4) Å^3^
*Z* = 4
Mo *K*α radiation
μ = 5.04 mm^−1^
*T* = 298 K
0.78 × 0.66 × 0.24 mm

#### Data collection

Bruker APEXII CCD diffractometer
Absorption correction: multi-scan (*SADABS*; Sheldrick, 2003 ▶) *T*_min_ = 0.259, *T*_max_ = 0.746
17190 measured reflections
3254 independent reflections
2281 reflections with *I* > 2σ(*I*)
*R*_int_ = 0.086

#### Refinement

*R*[*F* ^2^ > 2σ(*F* ^2^)] = 0.049
*wR*(*F* ^2^) = 0.132
*S* = 1.06
3254 reflections
167 parameters
H-atom parameters constrained
Δρ_max_ = 0.85 e Å^−3^
Δρ_min_ = −1.05 e Å^−3^
Absolute structure: Flack (1983 ▶), 1380 Friedel pairs
Flack parameter: 0.07 (2)

### 

Data collection: *APEX2* (Bruker, 2009 ▶); cell refinement: *SAINT-Plus* (Bruker, 2009 ▶); data reduction: *SAINT-Plus*; program(s) used to solve structure: *SHELXS97* (Sheldrick, 2008 ▶); program(s) used to refine structure: *SHELXL97* (Sheldrick, 2008 ▶); molecular graphics: *ORTEP-3 for Windows* (Farrugia, 1997 ▶); software used to prepare material for publication: *WinGX* (Farrugia, 1999 ▶).

## Supplementary Material

Crystal structure: contains datablock(s) I, global. DOI: 10.1107/S1600536812032333/im2394sup1.cif

Structure factors: contains datablock(s) I. DOI: 10.1107/S1600536812032333/im2394Isup2.hkl

Supplementary material file. DOI: 10.1107/S1600536812032333/im2394Isup3.cml

Additional supplementary materials:  crystallographic information; 3D view; checkCIF report

## Figures and Tables

**Fig. 1.**
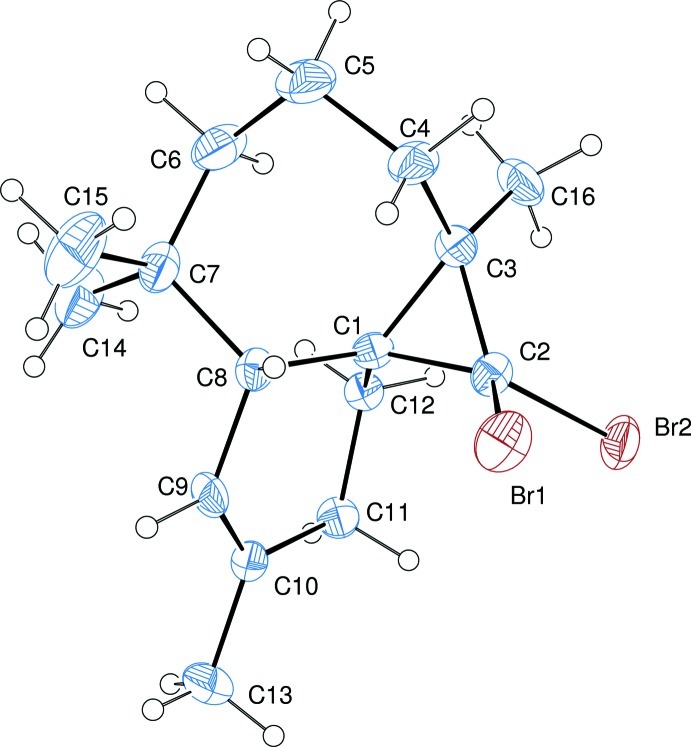
: Molecular structure of the title compound with displacement ellipsoids drawn at the 30% probability. level. H atoms are represented as small spheres of arbitrary radii.

## supplementary crystallographic information

### Comment

The bicyclic sesquiterpene β-himachalene is the main constituent of the essential oil of the Atlas cedar (Cedrus atlantica) (Plattier & Teiseire, 1974); Joseph & Dev, 1968). The reactivity of this sesquiterpene and its derivatives has been studied extensively by our team in order to prepare new products having biological proprieties. Lassaba et al., 1997; Chekroun et al., 2000; El Jamili et al., 2002; Sbai et al., 2002; Dakir et al., 2004). Indeed, these compounds were tested, using the food poisoning technique, for their potential antifungal activity against phytopathogen Botrytis cinerea (Daoubi et al., 2004). Thus the action of one equivalent of dibromocarbene, generated in situ from bromoform in the presence of sodium hydroxide as base and n-benzyltriethylammonium chloride as catalyst, on β-himachalene produces the title compound (I) with a yield of 22%. The structure of this new product was determined by its single-crystal X-ray structure analysis. The molecule is built up from two fused six-and seven- membered rings and an additional three-membered ring from the reaction with the carbene (Fig.1). The six-membered ring has a screw boat conformation, as indicated by the total puckering amplitude QT = 0.485 (19) Å and spherical polar angle θ = 128.1 (11)° with φ = 155.7 (14)°, whereas the seven-membered ring displays a boat conformation with QT = 1.1497 (1) Å, θ = 88.51 (5)°, φ2 = 311.8 (5)° and φ3 = 238.26 (19)° (Cremer & Pople, 1975). Owing to the presence of Br atoms, the absolute configuration could be fully confirmed, by refining the Flack parameter (Flack, 1983) as C1(S), C3(R) and C8(R).

### Experimental

A solution containing 5 g (24 mmol) of β-himachalene and 3 ml (37 mmol) of CHBr_3_ in 30 ml of dichloromethane was added in dropwise fashion at 0°C over 30 min to 1,5 g (37 mmol) of pulverized sodium hydroxide and 50 mg of N– benzyltriethylammonium chloride placed in a 100 ml three – necked flask. After stirring at room temperature for 2 h, the mixture was filtered on celite and concentrated in vacuum. The residue obtained was chromatographed on silica gel using hexane as eluting agent to give 2 g (5,3 mmol) of (1*S*,3*R*,8*R*)-2,2-Dibromo-3,7,7,10-tetramethyl-tricyclo [6.4.0.0^1,3^]dodec-9-ene with a yield of 22%. The title compound was recrystallized from pentane.

### Refinement

All H atoms were fixed geometrically and treated as riding with C—H = 0.96 Å (methyl), 0.97 Å (methylene), 0.98 Å (methine) and with *U*_iso_(H) = 1.2 *U*_eq_(C) for methylene and methine hydrogen atoms or *U*_iso_(H) = 1.5 *U*_eq_(C) for methyl groups.

### Crystal data

**Table:**

C_16_H_24_Br_2_	*F*(000) = 760
*M**_r_* = 376.17	*D*_x_ = 1.557 Mg m^−^^3^
Orthorhombic, *P*2_1_2_1_2_1_	Mo *K*α radiation, λ = 0.71073 Å
Hall symbol: P 2ac 2ab	Cell parameters from 17190 reflections
*a* = 9.7464 (14) Å	θ = 2.6–26.4°
*b* = 12.1633 (16) Å	µ = 5.04 mm^−^^1^
*c* = 13.5352 (18) Å	*T* = 298 K
*V* = 1604.6 (4) Å^3^	Prism, colourless
*Z* = 4	0.78 × 0.66 × 0.24 mm

### Data collection

**Table:**

Bruker APEXII CCD diffractometer	3254 independent reflections
Radiation source: fine-focus sealed tube	2281 reflections with *I* > 2σ(*I*)
Graphite monochromator	*R*_int_ = 0.086
ω and φ scans	θ_max_ = 26.4°, θ_min_ = 2.6°
Absorption correction: multi-scan (*SADABS*; Sheldrick, 2003)	*h* = −12→12
*T*_min_ = 0.259, *T*_max_ = 0.746	*k* = −15→15
17190 measured reflections	*l* = −16→16

### Refinement

**Table:**

Refinement on *F*^2^	Secondary atom site location: difference Fourier map
Least-squares matrix: full	Hydrogen site location: inferred from neighbouring sites
*R*[*F*^2^ > 2σ(*F*^2^)] = 0.049	H-atom parameters constrained
*wR*(*F*^2^) = 0.132	*w* = 1/[σ^2^(*F*_o_^2^) + (0.0682*P*)^2^ + 0.6114*P*] where *P* = (*F*_o_^2^ + 2*F*_c_^2^)/3
*S* = 1.06	(Δ/σ)_max_ < 0.001
3254 reflections	Δρ_max_ = 0.85 e Å^−^^3^
167 parameters	Δρ_min_ = −1.05 e Å^−^^3^
0 restraints	Absolute structure: Flack (1983), 1380 Friedel pairs
Primary atom site location: structure-invariant direct methods	Flack parameter: 0.07 (2)

### Special details

**Table:**

Geometry. All s.u.'s (except the s.u. in the dihedral angle between two l.s. planes) are estimated using the full covariance matrix. The cell s.u.'s are taken into account individually in the estimation of s.u.'s in distances, angles and torsion angles; correlations between s.u.'s in cell parameters are only used when they are defined by crystal symmetry. An approximate (isotropic) treatment of cell s.u.'s is used for estimating s.u.'s involving l.s. planes.
Refinement. Refinement of *F*^2^ against ALL reflections. The weighted *R*-factor *wR* and goodness of fit *S* are based on *F*^2^, conventional *R*-factors *R* are based on *F*, with *F* set to zero for negative *F*^2^. The threshold expression of *F*^2^ > 2σ(*F*^2^) is used only for calculating *R*-factors(gt) *etc*. and is not relevant to the choice of reflections for refinement. *R*-factors based on *F*^2^ are statistically about twice as large as those based on *F*, and *R*- factors based on ALL data will be even larger.

### Fractional atomic coordinates and isotropic or equivalent isotropic displacement parameters (Å^2^)

**Table:**

	*x*	*y*	*z*	*U*_iso_*/*U*_eq_	
C1	0.3347 (7)	0.6855 (4)	0.2295 (4)	0.0321 (13)	
C2	0.4162 (7)	0.7675 (5)	0.1685 (5)	0.0368 (15)	
C3	0.3869 (8)	0.6562 (5)	0.1251 (5)	0.0413 (15)	
C4	0.2801 (8)	0.6488 (6)	0.0455 (5)	0.0486 (19)	
H4A	0.3251	0.6479	−0.0185	0.058*	
H4B	0.2221	0.7135	0.0482	0.058*	
C5	0.1901 (11)	0.5455 (7)	0.0554 (6)	0.067 (2)	
H5A	0.2369	0.4842	0.0243	0.080*	
H5B	0.1049	0.5575	0.0199	0.080*	
C6	0.1559 (10)	0.5140 (6)	0.1633 (6)	0.062 (2)	
H6A	0.2404	0.4906	0.1948	0.074*	
H6B	0.0953	0.4507	0.1616	0.074*	
C7	0.0886 (7)	0.6022 (6)	0.2302 (5)	0.0480 (17)	
C8	0.1814 (6)	0.7070 (5)	0.2427 (4)	0.0340 (13)	
H8	0.1550	0.7576	0.1897	0.041*	
C9	0.1592 (8)	0.7681 (5)	0.3381 (5)	0.0457 (17)	
H9	0.0743	0.8016	0.3471	0.055*	
C10	0.2497 (7)	0.7784 (5)	0.4100 (5)	0.0424 (17)	
C11	0.3862 (7)	0.7238 (5)	0.4053 (4)	0.0399 (16)	
H11A	0.4029	0.6870	0.4678	0.048*	
H11B	0.4561	0.7798	0.3974	0.048*	
C12	0.4011 (8)	0.6409 (4)	0.3229 (4)	0.0349 (14)	
H12A	0.3576	0.5723	0.3417	0.042*	
H12B	0.4976	0.6265	0.3109	0.042*	
C13	0.2240 (10)	0.8474 (8)	0.4997 (7)	0.075 (3)	
H13A	0.1371	0.8839	0.4932	0.113*	
H13B	0.2955	0.9013	0.5060	0.113*	
H13C	0.2229	0.8014	0.5573	0.113*	
C14	0.0598 (10)	0.5426 (7)	0.3284 (7)	0.072 (3)	
H14A	0.0060	0.4779	0.3159	0.108*	
H14B	0.0103	0.5907	0.3718	0.108*	
H14C	0.1451	0.5219	0.3586	0.108*	
C15	−0.0479 (10)	0.6373 (8)	0.1882 (9)	0.092 (3)	
H15A	−0.0345	0.6672	0.1232	0.138*	
H15B	−0.0880	0.6923	0.2301	0.138*	
H15C	−0.1079	0.5749	0.1844	0.138*	
C16	0.5021 (8)	0.5748 (6)	0.1134 (6)	0.058 (2)	
H16A	0.5482	0.5876	0.0518	0.088*	
H16B	0.4656	0.5015	0.1142	0.088*	
H16C	0.5660	0.5834	0.1668	0.088*	
Br1	0.33615 (9)	0.89924 (6)	0.11733 (6)	0.0589 (3)	
Br2	0.60263 (7)	0.80174 (6)	0.20570 (6)	0.0556 (2)	

### Atomic displacement parameters (Å^2^)

**Table:**

	*U*^11^	*U*^22^	*U*^33^	*U*^12^	*U*^13^	*U*^23^
C1	0.037 (3)	0.027 (3)	0.033 (3)	0.000 (3)	−0.005 (3)	0.001 (2)
C2	0.039 (4)	0.036 (3)	0.035 (3)	−0.004 (3)	−0.003 (3)	0.006 (2)
C3	0.045 (4)	0.043 (3)	0.036 (3)	−0.005 (3)	0.003 (4)	−0.002 (3)
C4	0.060 (6)	0.056 (4)	0.030 (3)	−0.016 (4)	−0.007 (4)	−0.002 (3)
C5	0.081 (7)	0.074 (5)	0.046 (4)	−0.020 (5)	−0.010 (5)	−0.015 (4)
C6	0.071 (6)	0.056 (4)	0.058 (4)	−0.021 (4)	−0.011 (5)	−0.006 (4)
C7	0.038 (4)	0.058 (4)	0.048 (4)	−0.012 (4)	−0.003 (4)	0.004 (3)
C8	0.028 (4)	0.040 (3)	0.034 (3)	0.000 (3)	−0.004 (3)	0.003 (2)
C9	0.031 (4)	0.041 (3)	0.065 (4)	0.007 (3)	0.017 (4)	−0.008 (3)
C10	0.039 (4)	0.048 (4)	0.040 (4)	−0.012 (3)	0.007 (3)	−0.007 (3)
C11	0.038 (4)	0.052 (4)	0.030 (3)	−0.001 (3)	−0.007 (3)	−0.002 (2)
C12	0.036 (4)	0.033 (3)	0.036 (3)	0.002 (3)	−0.001 (3)	0.002 (2)
C13	0.067 (6)	0.089 (6)	0.070 (6)	−0.011 (5)	0.019 (5)	−0.045 (5)
C14	0.063 (6)	0.076 (5)	0.077 (6)	−0.025 (4)	−0.004 (5)	0.013 (4)
C15	0.051 (6)	0.099 (7)	0.127 (9)	−0.014 (5)	−0.034 (7)	0.009 (6)
C16	0.059 (5)	0.057 (4)	0.060 (5)	0.009 (4)	0.023 (4)	−0.012 (4)
Br1	0.0687 (6)	0.0419 (3)	0.0662 (5)	−0.0017 (4)	−0.0037 (4)	0.0198 (3)
Br2	0.0369 (4)	0.0639 (4)	0.0661 (5)	−0.0149 (3)	0.0047 (4)	0.0019 (4)

### Geometric parameters (Å, º)

**Table:**

C1—C2	1.519 (8)	C8—H8	0.9800
C1—C12	1.520 (8)	C9—C10	1.319 (10)
C1—C8	1.527 (9)	C9—H9	0.9300
C1—C3	1.545 (9)	C10—C11	1.488 (10)
C2—C3	1.503 (8)	C10—C13	1.497 (9)
C2—Br1	1.913 (6)	C11—C12	1.510 (8)
C2—Br2	1.931 (7)	C11—H11A	0.9700
C3—C4	1.501 (10)	C11—H11B	0.9700
C3—C16	1.505 (10)	C12—H12A	0.9700
C4—C5	1.539 (11)	C12—H12B	0.9700
C4—H4A	0.9700	C13—H13A	0.9600
C4—H4B	0.9700	C13—H13B	0.9600
C5—C6	1.546 (12)	C13—H13C	0.9600
C5—H5A	0.9700	C14—H14A	0.9600
C5—H5B	0.9700	C14—H14B	0.9600
C6—C7	1.550 (11)	C14—H14C	0.9600
C6—H6A	0.9700	C15—H15A	0.9600
C6—H6B	0.9700	C15—H15B	0.9600
C7—C15	1.508 (12)	C15—H15C	0.9600
C7—C14	1.539 (11)	C16—H16A	0.9600
C7—C8	1.573 (9)	C16—H16B	0.9600
C8—C9	1.506 (9)	C16—H16C	0.9600
			
C2—C1—C12	117.6 (6)	C9—C8—H8	106.2
C2—C1—C8	117.5 (5)	C1—C8—H8	106.2
C12—C1—C8	112.4 (5)	C7—C8—H8	106.2
C2—C1—C3	58.8 (4)	C10—C9—C8	125.7 (6)
C12—C1—C3	122.6 (5)	C10—C9—H9	117.2
C8—C1—C3	118.0 (5)	C8—C9—H9	117.2
C3—C2—C1	61.5 (4)	C9—C10—C11	121.6 (6)
C3—C2—Br1	122.4 (5)	C9—C10—C13	122.6 (7)
C1—C2—Br1	122.2 (5)	C11—C10—C13	115.7 (7)
C3—C2—Br2	118.4 (5)	C10—C11—C12	114.6 (6)
C1—C2—Br2	119.5 (4)	C10—C11—H11A	108.6
Br1—C2—Br2	107.3 (3)	C12—C11—H11A	108.6
C4—C3—C2	117.8 (6)	C10—C11—H11B	108.6
C4—C3—C16	113.8 (6)	C12—C11—H11B	108.6
C2—C3—C16	119.4 (7)	H11A—C11—H11B	107.6
C4—C3—C1	116.3 (6)	C11—C12—C1	109.6 (5)
C2—C3—C1	59.8 (4)	C11—C12—H12A	109.8
C16—C3—C1	119.6 (6)	C1—C12—H12A	109.8
C3—C4—C5	112.4 (6)	C11—C12—H12B	109.8
C3—C4—H4A	109.1	C1—C12—H12B	109.8
C5—C4—H4A	109.1	H12A—C12—H12B	108.2
C3—C4—H4B	109.1	C10—C13—H13A	109.5
C5—C4—H4B	109.1	C10—C13—H13B	109.5
H4A—C4—H4B	107.8	H13A—C13—H13B	109.5
C4—C5—C6	114.1 (6)	C10—C13—H13C	109.5
C4—C5—H5A	108.7	H13A—C13—H13C	109.5
C6—C5—H5A	108.7	H13B—C13—H13C	109.5
C4—C5—H5B	108.7	C7—C14—H14A	109.5
C6—C5—H5B	108.7	C7—C14—H14B	109.5
H5A—C5—H5B	107.6	H14A—C14—H14B	109.5
C5—C6—C7	118.2 (7)	C7—C14—H14C	109.5
C5—C6—H6A	107.8	H14A—C14—H14C	109.5
C7—C6—H6A	107.8	H14B—C14—H14C	109.5
C5—C6—H6B	107.8	C7—C15—H15A	109.5
C7—C6—H6B	107.8	C7—C15—H15B	109.5
H6A—C6—H6B	107.1	H15A—C15—H15B	109.5
C15—C7—C14	107.4 (8)	C7—C15—H15C	109.5
C15—C7—C6	110.4 (7)	H15A—C15—H15C	109.5
C14—C7—C6	104.8 (6)	H15B—C15—H15C	109.5
C15—C7—C8	108.6 (6)	C3—C16—H16A	109.5
C14—C7—C8	113.2 (6)	C3—C16—H16B	109.5
C6—C7—C8	112.3 (6)	H16A—C16—H16B	109.5
C9—C8—C1	109.0 (5)	C3—C16—H16C	109.5
C9—C8—C7	114.2 (5)	H16A—C16—H16C	109.5
C1—C8—C7	114.3 (5)	H16B—C16—H16C	109.5
